# Acute Multiple In-Stent Thromboses in a Patient With Clopidogrel-Fluconazole Interaction

**DOI:** 10.7759/cureus.23718

**Published:** 2022-03-31

**Authors:** Antoine El Khoury, Zela Maria Butchakdjian, Elham Lagha, Peter Semaan, Maroun Soueidy

**Affiliations:** 1 Cardiology, University of Balamand, Beirut, LBN; 2 Internal Medicine, Lebanese University, Beirut, LBN; 3 Internal Medicine, University of Balamand, Beirut, LBN; 4 Interventional Cardiology, Mount Lebanon Hospital, Hazmiyeh, LBN

**Keywords:** fluconazole, clopidogrel, coronary stent thrombosis, drug interaction, myocardial infarction

## Abstract

Clopidogrel is an anti-platelet that exerts its function by selectively inhibiting the binding of adenosine di-phosphate (ADP) to the P2Y12 receptor. Fluconazole is a fungistatic agent that alters fungal cell membranes. Both of these drugs act on the cytochrome P450 2C19. We report the case of an 83-year-old male that presented two days following coronary angioplasty with stent thrombosis, following the concomitant use of clopidogrel and fluconazole. We aim to study the interaction between clopidogrel and fluconazole. We hypothesize that fluconazole decreases the therapeutic level of clopidogrel, requiring an increase in dosage to achieve the same anti-thrombotic effect.

## Introduction

Clopidogrel exerts its anti-platelet function by the irreversible binding of its active metabolite to the P2Y12 receptor, thus inhibiting the binding of adenosine di-phosphate (ADP) to the latter. This failure of binding of ADP hinders platelet activation and aggregation [1]. Cytochrome P2C19 is part of the larger family of cytochrome P450 enzymes, which metabolize lipids, hormones, toxins, and drugs. Among other classes of drugs, CYP2C19 metabolizes clopidogrel [1] and fluconazole, which is an antifungal whose mechanism of action involves inhibition of this cytochrome [2]. CYP2C19 gene polymorphism affects the level of drug metabolites; thus, a reduced function leads to lower levels of metabolites, consequently needing higher amounts of clopidogrel to achieve the therapeutic level required by the patient [3]. Similarly, drugs that work simultaneously on this same cytochrome mimic these gene variants and lead to reduced efficacy, hence, requiring dose adjustments as well [3]. Therefore, co-administration of fluconazole and clopidogrel requires dose adjustment of clopidogrel since both of these drugs act on the same cytochrome.

## Case presentation

**Figure 1:**
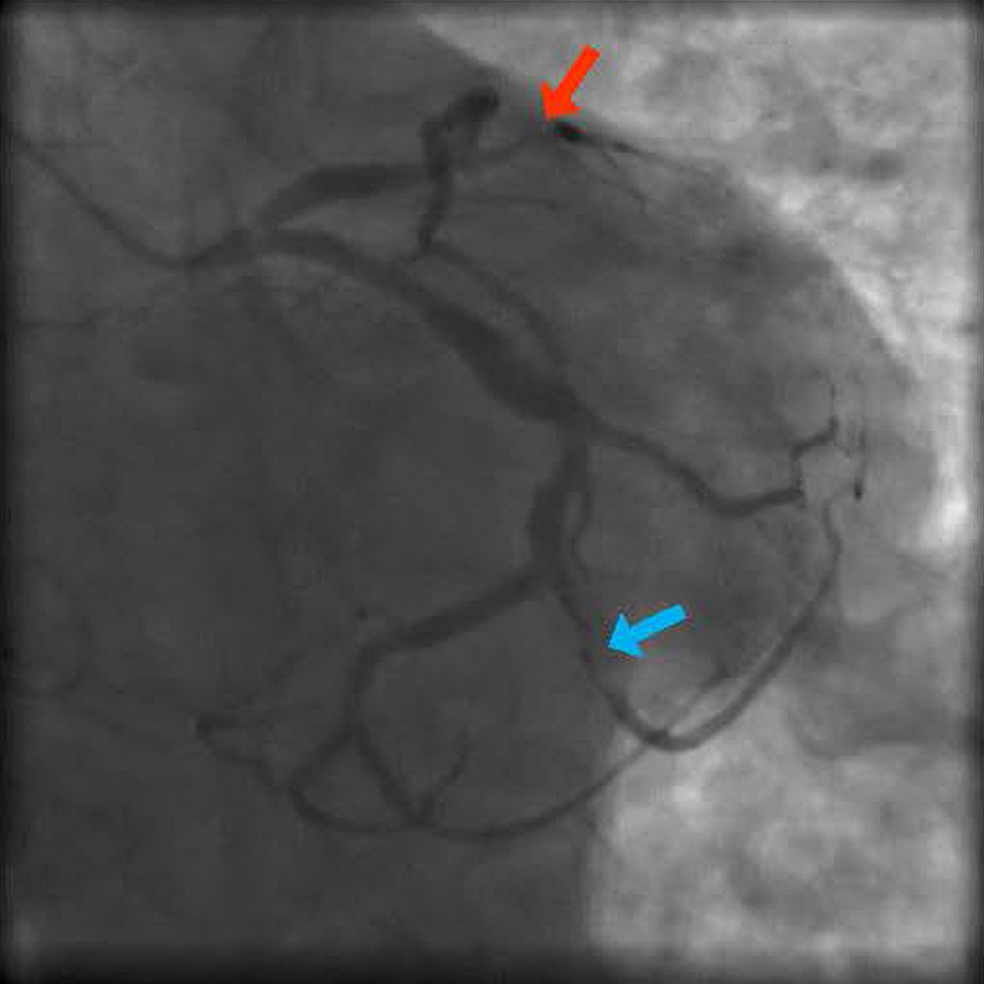
Multiple LAD lesions and severe lesions of the OM2 branch (LAD is marked by red arrow while OM2 is marked by blue arrow). LAD: left anterior descending artery; OM2: second obtuse marginal branch

**Figure 2:**
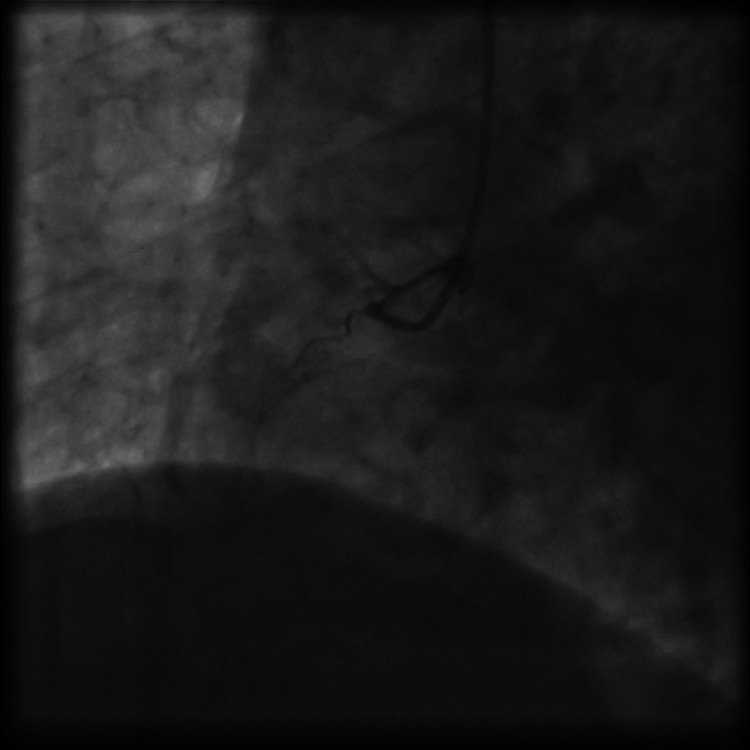
Chronic thrombosis of the RCA. RCA: right coronary artery.

**Figure 3:**
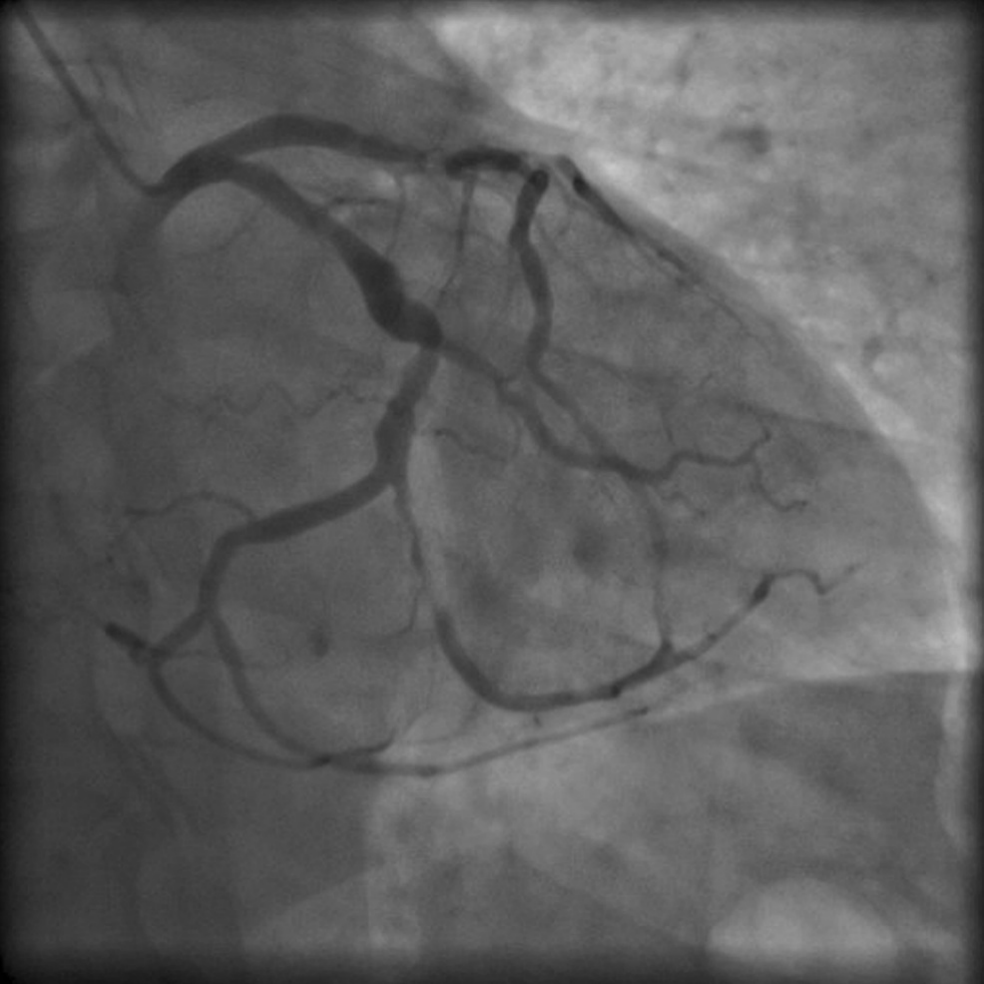
Result of the angioplasty of the LAD I/ LAD II and OM2 branch, with three stents after pre-dilatation with a non-compliant balloon. LAD I: left anterior descending artery first segment; LAD II: left anterior descending artery second segment; OM2: second obtuse marginal artery.

An 83-year-old male with a long history of diabetes and multiple ischemic cerebrovascular accidents presented for chest pain and unstable angina. Urgent coronary angiography done showed three-vessel disease with multiple lesions affecting the left anterior descending artery, a focal lesion affecting the obtuse marginal branch of the left circumflex artery, with chronic total occlusion of the right coronary artery (Figure 1 and Figure 2). Percutaneous balloon angioplasty was performed on the distal left anterior descending artery (LAD) and three everolimus drug-eluting stents were placed in the proximal LAD, mid-LAD, and obtuse marginal branch with good results and without complications (Figure 3).

**Figure 4:**
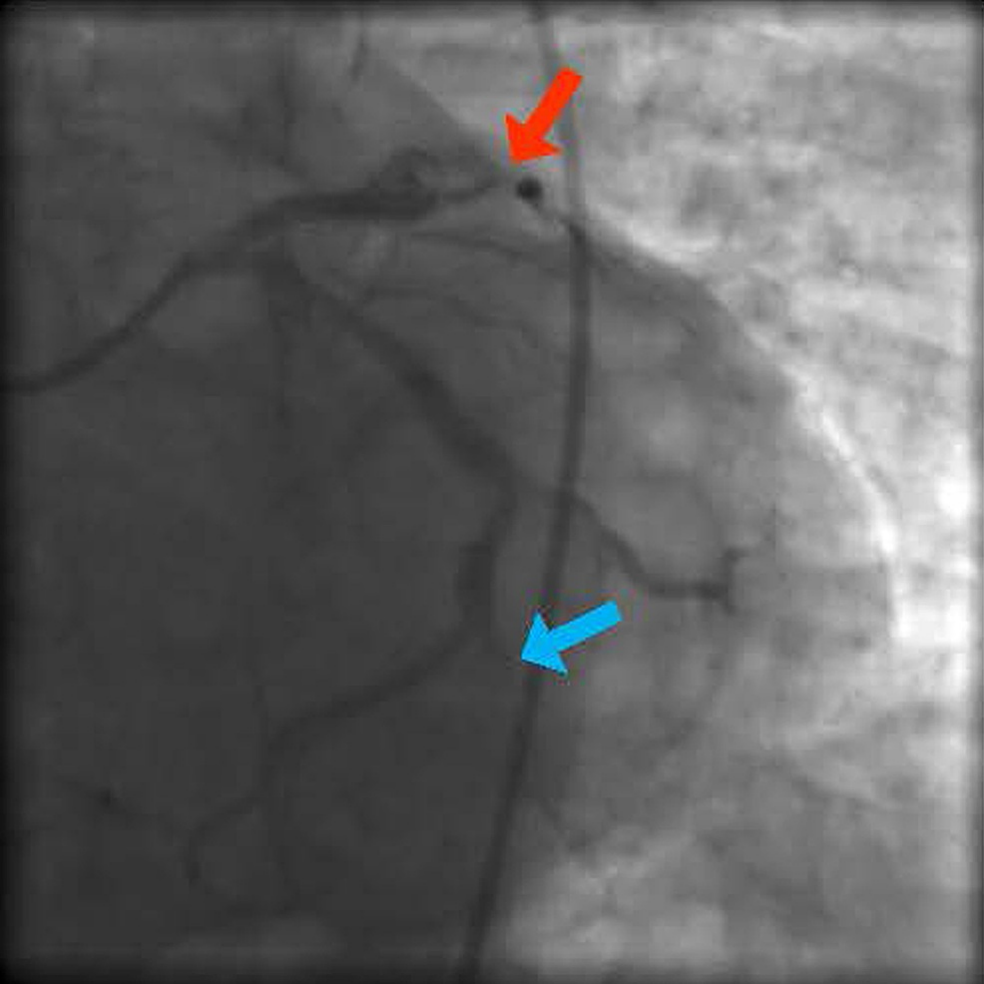
Acute re-thrombosis of the LAD and OM arteries (left anterior descending artery is marked by red arrow while obtuse marginal artery is marked by blue arrow). LAD: left anterior descending artery; OM: obtuse marginal artery

**Figure 5:**
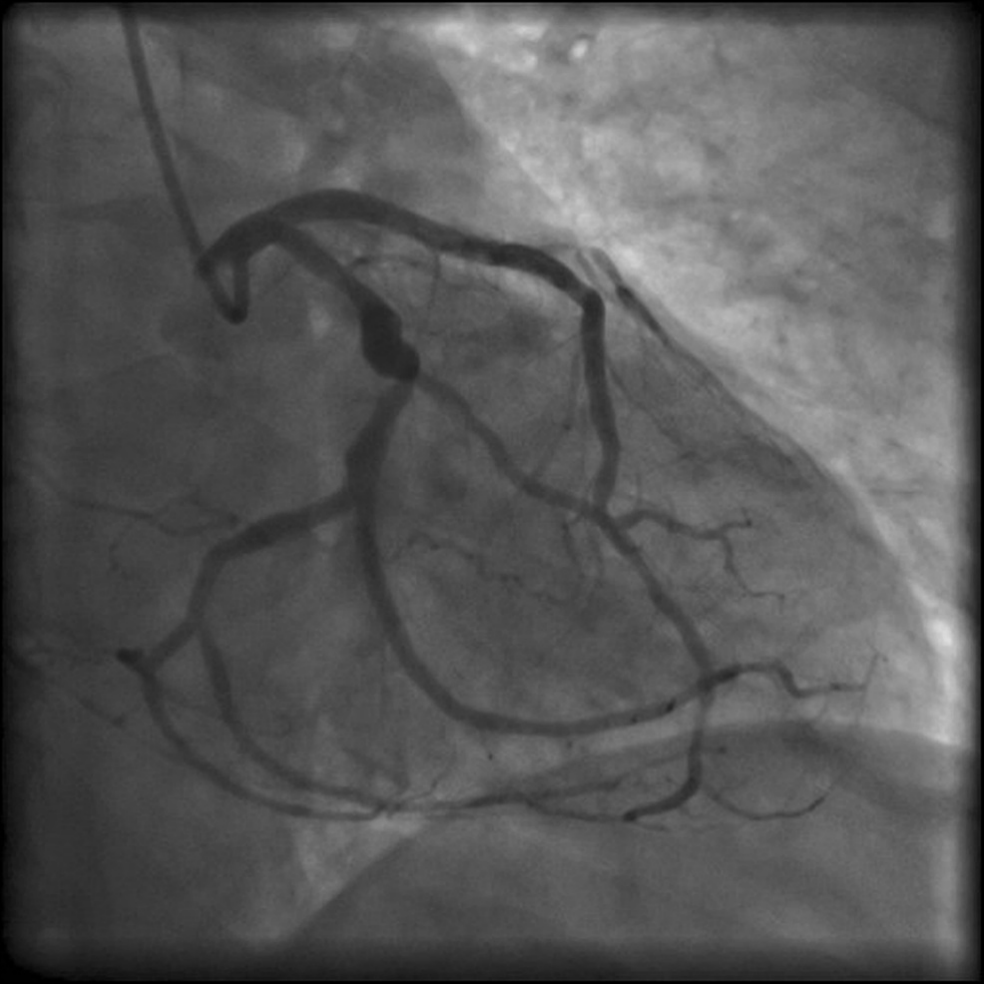
Successful recanalization of the LAD with positioning of three DESs and of the OM2 with one DES. LAD: left anterior descending artery; DES: drug-eluting stent; OM2: second obtuse marginal artery

The patient was placed on dual antiplatelet therapy: aspirin and clopidogrel, aggressive lipid-lowering agents, rabeprazole, and a diabetes control regimen. The patient was discharged home two days later with good adherence to therapy. Two days following discharge, the patient developed oral candidiasis. Of note, the patient has a history of oral candidiasis and thus self-medicated with oral fluconazole without seeking any medical advice. Two days later, and four days after the initial angioplasty, the patient presented to the emergency room with severe substernal chest pain radiating to the left arm and jaw, compatible with typical coronary artery disease (CAD) ischemic pain. The patient was found to have inferior and anterior ST-elevation myocardial infarction. He was then started on tirofiban and an urgent coronary angiography was performed, showing acute thrombosis at the level of the proximal and mid-LAD stents, mainly at the junction of both stents, and acute thrombosis of the obtuse marginal stent (Figure 4). Three drug-eluting stents were successfully placed on the LAD, in addition to a stent at the level of the thrombosed obtuse marginal branch (Figure 5).

Echocardiography done revealed inferior and anterior septal hypokinesia with an ejection fraction of 40%. The patient was stable post-procedure and was discharged home one week later. A resistance or drug interaction with clopidogrel was suspected and the patient was switched to ticagrelor. There was no recurrence of the disease and no new ischemic events in the next four years. 

## Discussion

Clopidogrel is an antithrombotic agent that acts on the cytochrome P2C19 in order to produce active metabolites to decrease platelet aggregation [1]. Decreased effectiveness of this drug is either linked to a mutation affecting the CYP2C19 leading to a different CYP2C19 genotype [4] and interaction with a different drug acting on this same cytochrome, a non-genuine product, or patient non-adherence to therapy [5]. Fluconazole, on the other hand, is a known inhibitor of CYP2C19 [2]. Having both agents act on this same cytochrome could potentially reduce the effectiveness of clopidogrel, leading to increased ischemic events. In consequence, clopidogrel dose adjustment might be necessary for patients placed on both these drugs simultaneously [3]. In patients with a loss of one allele of the CYP2C19, an increase in the clopidogrel dose is advised. A loading dose of 600 mg replaces the usual 300 mg dose, and a maintenance dose of 150 mg is administered instead of 75 mg [3].

Different antithrombotic agents such as ticagrelor or prasugrel could be alternatively used in such cases, owing to the fact that they both inhibit platelet aggregation without interacting with CYP2C19 [6,7]. According to the European Society of Cardiology (ESC) guidelines for the management of acute coronary syndromes in patients presenting without persistent ST-segment elevation, both these agents are the preferred P2Y12 inhibitors in the management of such cases, and clopidogrel is considered class IC [8]. However, these drugs were not available in our country at that time so the patient was alternatively prescribed clopidogrel.

Our patient was adherent to medical therapy post-discharge two days prior to the acute in-stent thrombotic event. The drug was thoroughly examined and verified to be genuine and not out of date. A genetic study to detect an alteration in the genotype was performed but the result was inconclusive. The initiation of fluconazole two days post-discharge for oral candidiasis was considered to be the direct trigger of the thrombotic event due to its interaction with clopidogrel. The patient was discharged on aspirin 100 mg and clopidogrel 75 mg, both doses were thus presumed not to be enough to reduce the risk of drug-drug interaction. The patient was also placed on rabeprazole, which is a proton pump inhibitor known to have less drug-drug interaction with clopidogrel than omeprazole [9]. Patients taking dual antiplatelet therapies should be placed on proton pump inhibitors in order to decrease gastrointestinal bleeding [8]. Thus, and owing to its high level of interaction with clopidogrel, the use of omeprazole was strictly avoided.

Fluconazole is well-known to decrease the effectiveness of clopidogrel [10]. To maintain the same antithrombotic efficacy, the clopidogrel dose should be increased or an antifungal from a different class than azoles - such as echinocandins, polyenes, or allylamines - should be used depending on the case [11-13]. This theory was highly suggested by acute thrombosis in all stents in all locations, which was in favor of a direct trigger, revealed to be the fluconazole-clopidogrel interaction. 

In addition to its interaction with inhibitory agents, clopidogrel also interacts with inducers such as St John’s wart, cyclosporine, angiotensin-converting enzyme inhibitors, aspirin, and curcumin. These drugs increase the effects of clopidogrel, leading to additional platelet inhibition and eventually to bleeding when used concomitantly [14].

The theory of interaction between fluconazole and everolimus found in the drug-eluting stent is not very supported due to the local effect of everolimus. In fact, everolimus is a mammalian target of rapamycin inhibitor (an m-TOR inhibitor) and does not act on the CYP2C19 [15]. Moreover, this interaction in the setting of coronary stents is non-existent. 

## Conclusions

Acute in-stent thrombosis in multiple stents at the same time should raise the possibility of antiplatelet drug resistance, mainly for patients using clopidogrel. A mutation of a CYP2C19 allele or a drug-drug interaction could trigger the thrombotic event. Drugs such as azole antifungals should be avoided with the use of clopidogrel, mainly in the early course after coronary stenting. In case it is necessary to use fungal agents, it is advised to double clopidogrel dosage or switch from clopidogrel to a different class of antiplatelet drugs such as prasugrel and ticagrelor that do not act on CYP2C19. The goal of our paper is to shed light on the importance of evaluating drug-drug interactions before prescribing any medication to patients. In the management of our patient, we avoided the use of omeprazole given its high interaction with clopidogrel. However, our patient self-medicated with fluconazole at home, a factor we could not control, leading to thrombosis of all four stents placed. Moreover, it is crucial for physicians to advise patients, especially ones with multiple comorbidities, on the importance of seeking medical advice before initiating any drug because it could lead to deleterious effects as seen in our case.

## References

[1] Wijeyeratne YD, Heptinstall S (2011). Anti-platelet therapy: ADP receptor antagonists. Br J Clin Pharmacol.

[2] Dymond AW, So K, Martin P (2017). Effects of cytochrome P450 (CYP3A4 and CYP2C19) inhibition and induction on the exposure of selumetinib, a MEK1/2 inhibitor, in healthy subjects: results from two clinical trials. Eur J Clin Pharmacol.

[3] Saab YB, Zeenny R, Ramadan WH (2015). Optimizing clopidogrel dose response: a new clinical algorithm comprising CYP2C19 pharmacogenetics and drug interactions. Ther Clin Risk Manag.

[4] Savi P, Nurden P, Nurden AT, Levy-Toledano S, Herbert JM (1998). Clopidogrel: a review of its mechanism of action. Platelets.

[5] Ma TK, Lam YY, Tan VP, Yan BP (2011). Variability in response to clopidogrel: how important are pharmacogenetics and drug interactions?. Br J Clin Pharmacol.

[6] Dobesh PP, Oestreich JH (2014). Ticagrelor: pharmacokinetics, pharmacodynamics, clinical efficacy, and safety. Pharmacotherapy.

[7] Mousa SA, Jeske WP, Fareed J (2010). Antiplatelet therapy prasugrel: a novel platelet ADP P2Y12 receptor antagonist. Clin Appl Thromb Hemost.

[8] Collet JP, Thiele H, Barbato E (2021). 2020 ESC guidelines for the management of acute coronary syndromes in patients presenting without persistent ST-segment elevation. Eur Heart J.

[9] Norgard NB, Mathews KD, Wall GC (2009). Drug-drug interaction between clopidogrel and the proton pump inhibitors. Ann Pharmacother.

[10] Anderson CD, Biffi A, Greenberg SM, Rosand J (2010). Personalized approaches to clopidogrel therapy: are we there yet?. Stroke.

[11] Sucher AJ, Chahine EB, Balcer HE (2009). Echinocandins: the newest class of antifungals. Ann Pharmacother.

[12] Serhan G, Stack CM, Perrone GG, Morton CO (2014). The polyene antifungals, amphotericin B and nystatin, cause cell death in Saccharomyces cerevisiae by a distinct mechanism to amphibian-derived antimicrobial peptides. Ann Clin Microbiol Antimicrob.

[13] Birnbaum JE (1990). Pharmacology of the allylamines. J Am Acad Dermatol.

[14] Wang ZY, Chen M, Zhu LL, Yu LS, Zeng S, Xiang MX, Zhou Q (2015). Pharmacokinetic drug interactions with clopidogrel: updated review and risk management in combination therapy. Ther Clin Risk Manag.

[15] Macaskill EJ, Bartlett JM, Sabine VS (2011). The mammalian target of rapamycin inhibitor everolimus (RAD001) in early breast cancer: results of a pre-operative study. Breast Cancer Res Treat.

